# Extended Reality for Enhancing Surgery: Research Directions to Transform the Operating Environment

**DOI:** 10.1007/s41666-026-00250-y

**Published:** 2026-08-11

**Authors:** Gabriel Zachmann, Peter Haddawy, Ron Kikinis, Anja Hennemuth, Philippe C. Cattin, Tanja Döring, Simon Drouin, Gabor Fichtinger, Tabea Flügge, Joaquim A. Jorge, Luv Kohli, Mario Lorenz, Rainer Malaka, Javier Pascau, Dirk Reiners, Anke V. Reinschluessel, Andrea Schenk, Falko Schmid, Siriwan Suebnukarn, Verena Uslar, Gregory F. Welch, Rene Weller, Dirk Weyhe, Myat Su Yin, Sudanthi Wijewickrema

**Affiliations:** 1 University of Bremen, Bremen, Germany; 2 Mahidol University, Nakhon Pathom, Thailand; 3 Harvard Medical School, Boston, USA; 4 Charité - Universitaetsmedizin, Berlin, Germany; 5 Universität Basel, Allschwil, Switzerland; 6 ETS, Montreal, Canada; 7 Queen’s University, Kingston, Canada; 8 INESC-ID, Instituto Superior Técnico da Universidade de Lisboa, Lisbon, Portugal; 9 InnerOptic Technology, Hillsborough, NC USA; 10 Professorship for Production Systems and Processes, Chemnitz University of Technology, Chemnitz, Germany; 11 Department of Orthopedics, Trauma and Plastic Surgery, University Hospital Leipzig, Leipzig, Germany; 12 Gottfried Schatz Research Center, Division of Macroscopic and Clinical Anatomy, Medical University of Graz, Graz, Austria; 13 Universidad Carlos III de Madrid ES, Madrid, Spain; 14 University of Central Florida, Orlando, USA; 15 University of Bayreuth, Bayreuth, Germany; 16 Fraunhofer MEVIS, Bremen, Germany; 17 Heinrich Heine Universitätsklinikum, Düsseldorf, Germany; 18 Thammasat University, Pathum Thani, Thailand; 19 Universität Oldenburg, Oldenburg, Germany; 20 Australian Centre for AI in Medical Innovation, La Trobe University, Melbourne, Australia; 21 Hannover Medical School, Hannover, Germany

**Keywords:** Extended reality, Artificial intelligence, Surgery, Multimodal rendering, Tissue modeling, Robotic surgery

## Abstract

In the past decade, virtual reality (VR) and augmented reality (AR) have seen a second wave of activity due to consumer-level availability of devices. In the medical realm, use of VR and AR has been investigated extensively and successfully for training, teaching, rehabilitation, and therapy. However, extended reality (XR) and its intraoperative use in the operating room has yet been underexplored and underdeveloped. Intelligent XR environments that integrate advanced visualization, intuitive interaction, and AI-assisted decision support have the potential to transform clinical care. Responsive, context-aware spaces could enhance medical teams’ capabilities by enabling real-time access to critical information, enhancing situation awareness, and supporting remote collaboration and access to clinical expertise across geographical boundaries—all while preserving the natural flow of procedures. But to realize the potential benefits of XR in the operating room requires a significant and focused effort by an interdisciplinary community. It is the intention of this paper to help to catalyze such an effort. In this paper, we identify pressing issues in clinical care, the potential of XR to address them, and the further research that is needed to realize that potential. We look at a broad range of areas where XR can play an important role, including direct support for the surgeon, support for the surgical team, support for the patient, and remote collaboration. We also consider ways in which XR can be integrated with AI to provide enhanced information and interaction. Discussions are underpinned by considerations of methods to evaluate contributions and progress. This paper is the result of a Dagstuhl Seminar on *Extended Reality for the Operating Room (XR4OR)*, held at the Leibniz Center for Informatics, Schloss Dagstuhl, Germany, in February 2025. It gathered twenty-four researchers working in the areas of virtual reality, augmented reality, medical informatics, human-computer interaction, and surgery.

## Introduction

In the past decade, virtual reality (VR) and augmented reality (AR) have seen a second wave of activity due to consumer-level availability of devices. The whole spectrum of Milgram’s Continuum [1], called Extended Reality (XR), is now widely available and continues to grow both technologically and scientifically. These technologies are transforming medical education, surgical planning, rehabilitation, and therapy [2, 3] by providing immersive and interactive experiences that enhance learning, precision, and patient outcomes.

In medical education and training, XR has demonstrated superiority over traditional methods such as cadaveric dissection, textbook learning, and 2D simulations by improving spatial understanding, procedural retention, and skill transferability. For instance, VR medical simulators enable trainees to practice complex procedures in a controlled environment, leading to quantifiable improvements in performance metrics [4, 5]. Studies consistently show that VR training enhances surgical skills and confidence, with trainees performing better in speed and accuracy compared to traditional training methods [6, 7].

In surgical planning, XR technology facilitates personalized 3D reconstructions from patient imaging, enabling surgeons to visualize complex anatomy and rehearse procedures with enhanced accuracy and efficiency [8, 9]. This capability is particularly valuable where precise spatial understanding is critical, decreasing operative time and blood loss.

But while XR has proven to improve certain aspects of clinical medicine, its intraoperative use in the operating room is still in its infancy. This is not only due to barriers such as regulatory hurdles and technology limitations, but also because the ways in which the technologies could be useful in the OR have yet been underexplored and underdeveloped. The promise and maturity of XR technology together with its potential impact and underexploration in surgery motivates this paper.

Intelligent XR environments that integrate advanced visualization, intuitive interaction, and AI-assisted decision support have the potential to transform surgical practice. Responsive, context-aware spaces could enhance medical teams’ capabilities by enabling real-time access to critical information, enhancing situation awareness, and supporting remote collaboration and access to clinical expertise across geographical boundaries—all while preserving the natural flow of procedures. With approximately 310 million major surgeries performed annually globally [10], even small improvements in surgical efficiency and outcomes through XR technology could have significant impacts on national healthcare.

**Fig. 1:**
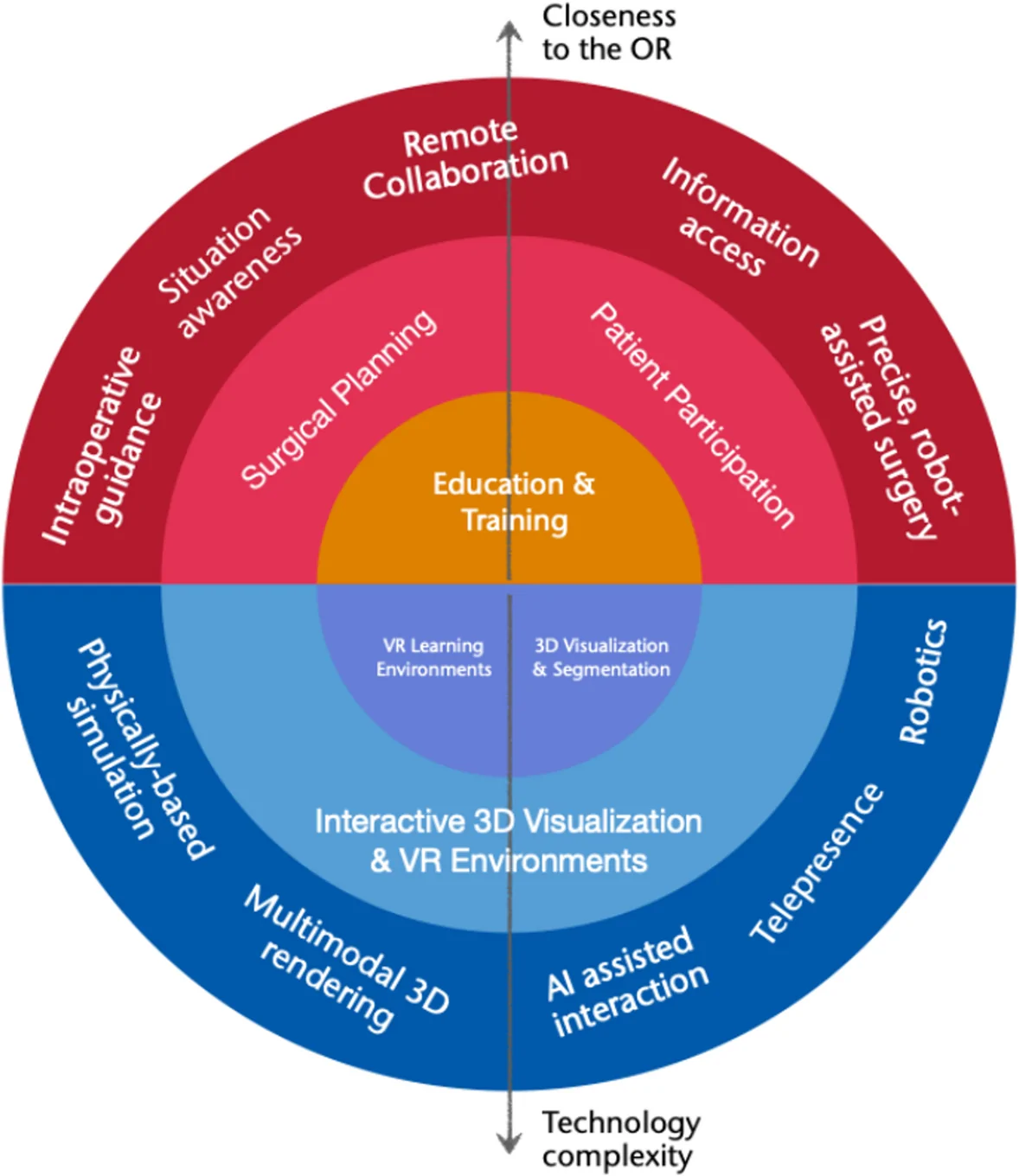
Needs and application areas of medical care (red) where XR technologies (blue) are currently being used or could be used in the future. In this paper, we focus mostly on the outer ring of applications and technologies (including “Patient participation”)

In this paper, we identify pressing issues in surgical practice, the potential of XR to address them, and the further research that is needed to realize that potential (see Fig. 1). The themes emerged from an in-depth discussion among clinicians and computer scientists in which key clinical challenges were identified and then appropriate technologies to address them were explored. The major challenges identified included: information overload in the OR, resulting in high cognitive load and lack of situation awareness; the accurate rendering of pre-operative medical images, particularly given the current surgical state; the non-uniform availability of surgical expertise across geographic regions; the need to facilitate minimally invasive surgery as a means to improve outcomes; and the need to increase the effectiveness of patient participation in decision making. Consideration of technologies to address these naturally gives rise to issues regarding robustness of the technology, as well as how to evaluate its effectiveness. This paper is thus organized around the following key themes which systematically address both the technical and human factors aspects of XR in surgery, while maintaining focus on practical clinical value and patient outcomes.

**Multimodal Rendering and Data Integration** examines how to present the right information from multiple data sources using various presentation modalities at the right time.

**Deformable Registration and Tissue Modeling** examines the problem of accurately simulating, tracking, and guiding interventions on soft tissues.

**Interaction and AI** investigates natural and efficient ways for medical teams to control XR systems and collaborate with AI, emphasizing the balance between automation and human control.

**Remote Collaboration** explores how XR can enable effective availability of expert surgeons and expert consultation.

**Robotics** examines the integration of XR with surgical robotics systems, focusing on control interfaces and feedback mechanisms.

**Surgeon Perspectives** discusses how XR can support specific needs of surgeons in terms of training, mentoring, intraoperative support, and work-life balance.

**Patient Perspectives** considers how XR can improve understanding, participative decision-making, and experience throughout the surgical treatment process.

**Robustness and Uncertainty** addresses the critical requirements for reliability, error management, and maintaining trust in XR-enhanced surgical systems.

**Evaluation** addresses the multi-dimensional question of measurement and evaluation of XR’s impact on surgical interventional outcomes, including parameters of the surgical procedure, socioeconomic considerations, patient quality of life, and ethical considerations.

This paper is a result of the Dagstuhl Seminar, *Extended Reality for the Operating Room (XR4OR)*,^1^ held at the Leibniz Center for Informatics, Schloss Dagstuhl, Germany, in February 2025. It gathered 24 researchers in the areas of virtual reality, augmented reality, medical informatics, human-computer interaction, and surgery. All participants worked in a structured process on the topic for four days at the seminar and collaborated online thereafter.

## Multimodal Rendering and Data Integration

This section explores the potential of Extended Reality technologies in enhancing surgical procedures by data integration and multimodal rendering techniques. The two topics are related since multimodal rendering often requires data to be integrated before they can be rendered. In terms of data integration, the section highlights the challenges of fragmented data streams and poor interoperability in operating rooms and discusses the potential of AR to address them. For multimodal rendering techniques, we discuss the challenges of visualizing complex 3D medical data in AR, including depth perception, occlusion management, and adaptive rendering techniques to handle varying lighting conditions and user-specific visual impairments. The goal is to improve surgical procedures by providing the right information at the right time and enhancing the visualization of critical anatomical and contextual information.

### Data Integration: The Right Information in the Right Place at the Right Time

#### Motivation

Operating rooms are full of devices (e.g., vital sign monitors, imaging systems) that often operate in isolation, leading to fragmented data streams and poor interoperability. Patient information can become siloed, leaving the burden on team members to filter important information and verbally communicate critical data in real time. This situation contributes to a high cognitive load for all stakeholders, carries the risk of miscommunication, and results in decisions being made based on potentially incomplete information. In this section, we investigate the added value of a future operating room where state-of-the-art technology for AR is available at all times. This approach, we believe, is likely to enable a more space- and information-efficient operating room.

At the time of this writing, the next generation of AR glasses (e.g. Meta Orion) gives a glimpse into the form factors that AR glasses will have in the near- and mid-term future. Meta’s Orion already will be light enough to allow users to wear them throughout a surgical procedure. In a scenario where each member of the surgical team wears a pair of such glasses, various pieces of large equipment, starting with the numerous display screens, could be removed from the operating room. Instead, the data collected from machines and sensors in the OR could be broadcast to all the glasses; each member of the team could compose a personalized virtual heads-up display, thus seeing only the relevant information. High-resolution AR glasses can overlay all kinds of information directly into the wearer’s field of view as a personalized, context-aware display. For instance, a surgeon might see anatomical holograms, while the anesthesiologist sees detailed vital trends and alarms; a scrub nurse’s view could highlight remaining instrument counts or timing for antibiotics. Important changes to the patient’s condition can be pushed to everyone’s display, reducing the need to verbally request information. AR interfaces can also improve focus and situational awareness – eliminating the time and mental effort spent glancing around at different monitors. A less cluttered OR, particularly in the first meter around the operating field, could have a positive impact on the surgical workflow, the cognitive load of the surgical team, and eventually the patient outcome.

#### State-of-the-Art

State-of-the-art commercial approaches for surgery support through integrated visualizations add monitors for the combined display of complementary data^2^. AR researchers have focused on simple integration of a few information sources, often for a single person (the surgeon) with the goal of reducing attention shifts. One example of this concept is the integration of ultrasound and endoscopic video feeds as virtual screens within HMDs, improving situational awareness [11]. Other research prototypes have demonstrated that an AR visor can pull in multiple video and data sources – e.g. endoscopic camera views, CT scans, and pre-op plans – and display them together in the headset, allowing the surgeon to access all critical information without looking away from the patient [12]. In the commercial field, the BrainLab system already integrates surgical microscopes and ultrasound machines into their workflow, and other data sources (imaging and image reconstruction) can be integrated into the proprietary workflow [13].

On the research side, one important initiative to enable device integration, though not specific to XR, is the adaptation of the open source Nebula Stream solution [14] to the integrated analysis of multiple clinical data streams^3^. Another one is the open source Plus Toolkit, using a dedicated wireless network [15]. For example, it is possible to run the PlusToolkit on a computer physically connected to an ultrasound machine, adjust the acquisition parameters (e.g. probe depth), then broadcast the captured images on the network to be displayed elsewhere. The Plus Toolkit uses an open and standardized protocol (OpenIGTLink) to connect devices, which supports various types of data (volumes, images, transforms, etc.). In the current version, the setup of an operating room with a large number of interconnected devices remains challenging, though. There is a need for either a centralized server, or a decentralized system where each device gets informed of all the other OR devices delivering information.

#### Future Research

Several research questions must be investigated before the AR based operating room can be implemented: (1) What are the desirable form factors and properties of the AR HMDs, (2) What kind of network infrastructure and protocols are needed to ensure efficient and safe flow of information, (3) How does the new technological infrastructure influence OR layout and personnel interaction and most importantly, (4) How to personalize information for every role (surgeon, anesthesiologist, nurse) so that each person receives the specific information they need without being overloaded by extraneous data. We focus on this last aspect for the remainder of this section.

Personalizing displays requires discriminating between role-specific and team relevant information. An AR system should filter information so each specialist can see the information relevant to their task, yet ensure that shared critical parameters are communicated to all necessary personnel. Striking this balance is key to avoiding information overload and making sure no critical information is left unnoticed. Furthermore, it should be possible for every member of the surgical team to reconfigure their displays in real time during the operation and share information about their assessment of the condition of the patient while making sure they can’t miss critical information because of such reconfiguration. These functionalities require a simple user interaction mechanism that takes into account sterility constraints, such as voice commands, hand gestures, and eye tracking. A first step in validating the proposed model for an all-AR operating room is to build a multi-user virtual reality simulator for such an OR. The simulator would enable rapid prototyping and validation for new ideas on alternative OR layouts, personalized AR displays as well as user interaction mechanisms.

### Multimodal Rendering Techniques and Presentation

#### Motivation

One goal of surgical AR is to enhance the surgeon’s understanding of critical anatomical and contextual information by integrating 3D rendered content with live imagery from the OR. The result should be a coherent visual field in which real and virtual elements are perceptually aligned, both in terms of spatial positioning and semantic relevance. Achieving such perceptual coherence, however, remains a significant challenge given the limitations of current hardware and rendering techniques.

A fundamental issue lies in the accurate portrayal of depth and the effective management of occlusion when visualizing complex 3D medical data. Current AR displays often struggle to convey the correct relative depth between virtual anatomical models and live images, which impairs the perception of shape and spatial arrangement. This limitation becomes more pronounced when the system attempts to enhance real anatomical structures—such as blood vessels—with virtual overlays derived from preoperative imaging or intraoperative sensing.

The fusion of real and virtual components is further complicated by discrepancies in lighting and color balance. For AR visualizations to remain perceptually stable and usable within the variable lighting conditions of an operating theater, the rendering system must dynamically adjust contrast and illumination to maintain visual clarity across both domains. Failure to do so can lead to misleading visual cues or reduced visibility of key information.

#### State-of-the-Art

Enabling physicians to see inside patients has been investigated for decades [16, 17]. Researchers have also explored using near-infrared light or multispectral imaging to segment organs and visualize cancer tissue [18, 19]. By displaying such visualizations, physicians can more easily ensure that they treat everything that requires treatment.

Several strategies have been proposed to facilitate depth perception of 3D rendered medical images, including encoding depth with color [20], filling void space with various depth cues [21], simulating aerial perspective [22], automatically positioning depth anchors [23], and rendering depth of focus. However, most of the techniques proposed so far have been validated only for 2D monitor displays. A comprehensive, if somewhat dated, review of depth-enhancement strategies can be found in [24]. Others have proposed to rely on user interaction to improve depth perception [25], based on findings from the human visual perception literature. Recent work suggests that head mounted displays with stereoscopic and parallax capabilities can limit the advantages of some of the depth enhancement techniques mentioned above.

Numerous solutions have been proposed to manage inter-occlusion of 3D rendered content, with the goal of revealing otherwise occluded structures of interest and their spatial relationship with the rest of the anatomy. Various volumetric clipping and cropping methods have been proposed [26], but they make it difficult to appreciate the three-dimensional structure of the anatomy. Interactive deformation frameworks let users push away occluding anatomical structures, but usually rely on the availability of a prior segmentation (see Sect. 3). Very few of these methods have been validated in the context of AR and interaction techniques with such methods remain a challenge in AR. For an in-depth review of occlusion management strategies with 3D rendering, refer to [27].

The visible surface of the operating field is either directly visible by the user (optical see-through AR) or captured using a camera on the AR device. This layer is usually a regular 2D image that is sometimes combined with an additional, low resolution depth channel. The simplest rendering technique is a semi-transparent overlaying the computer-generated imagery, giving the impression that every pixel of the image is closer to the observer than the patient’s internal anatomy. This approach could lead to an incorrect perception of depth where virtual 3D content appears to be floating above the surface of the operating field. One common strategy to solve this problem is to introduce occlusion cues by modulating the opacity of the image [28].

The third layer contains different objects like surgical tools and the hands of the clinicians. Although these elements are usually captured with the same camera as layer 2, they demand a special treatment as they must be depicted as opaque to avoid introducing confusing occlusion cues. For an in-depth review of the occlusion management strategies in the context of AR, refer to [29].

#### Future Research

While several solutions have been proposed to solve the perceptual issues posed by augmented reality, these are rarely integrated in devices and software such that they can be validated by clinicians [24]. In a typical OR scenario, an AR view should be able to overlay three distinct layers of information: (1) A patient’s internal anatomy of interest to the procedure, (2) The visible surface of the operating field, (3) The surgeon’s hands and the surgical tools. Each of these layers present depth perception and occlusion challenges that need to be solved.

Further research is also needed on rendering personalization. Visualization systems should be capable of dynamically adjusting to ambient lighting, visual impairments (e.g., color blindness), and the dominant colors already present in the operating field. These adaptations would enhance visibility and reduce perceptual strain, ensuring that virtual overlays remain effective and accessible across a diverse user base and in varying environmental conditions.

While surgical AR holds considerable promise for enriching intraoperative perception and decision-making, several core challenges must be addressed. These include the reliable management of depth and occlusion in mixed reality scenes, the seamless blending of real and virtual components, adaptive lighting and contrast adjustment, and the contextual presentation of dynamic patient and procedural data.

## Deformable Registration and Tissue Modeling

In this section, we discuss challenges and opportunities of intraoperative simulation and guidance, which includes tracking and registration issues. We highlight the need for robust, accurate, and fast models to simulate complex tissue behaviors under varying physiological conditions. The focus is on improving the precision and safety of surgical procedures through advanced simulation and modeling techniques.

### Motivation

In soft tissue interventions, the ability to accurately simulate, track, and guide interventions on those tissues presents both substantial challenges and opportunities in various applications, such as preoperative planning, intra operative guidance, and training. In preoperative planning, the surgeon needs to anticipate the behavior of tissues under different conditions, such as a pressure change in the brain following a dural incision, or the change of shape of an organ during the procedure. Similar situations arise in the case of organ-preserving tumor resections, where the relative locations of both the tumor and underlying vascular structure needs to be predicted. Simulating such complex tissue dynamics could significantly enhance a surgeon’s understanding of the risks before the procedure begins.

During surgery, challenges include the precise localization and navigation of instruments in close proximity to anatomical structures, such as blood vessels, nerves, or neuro-functional regions of the brain. Precise soft tissue simulations would be of great benefit here, particularly for generating visualizations in limited-view situations. Such simulations could alert the team when a critical structure might be endangered. Not only could this prevent damage, but also allow for more precise placement of stents or implants. Furthermore, such simulations could greatly benefit robotically-assisted surgeries. For other kinds of therapies, such as radiation therapy, soft tissue simulation could help with tracking radiation necrosis, inflammation, and tumor shrinkage, etc. For training, such simulations can provide realistic models of organ behavior during surgery, including haptic feedback, thus allowing for better learning of surgical skills.

While deformable tissue modeling, tracking, and navigation have improved over the past decades, significant research and development efforts are still necessary. There is a critical need for more robust, accurate, fast models that simulate complex soft tissue behaviors under varying physiological conditions. A particularly difficult challenge is the automatic acquisition of the parameters of the models, such as elasticity; and this is even more difficult to achieve for patient-specific models. However, great benefits are likely to be achieved, in terms of safer, more efficient, and better personalized treatments for patients.

### State-of-the-Art

Deformable registration of 3D models for minimally invasive or open surgery has been the subject of many years of research with still unsatisfactory results. Depending on the imaging technology, it is performed in the OR as 2D-3D or 3D-3D registration [30]. Several methods have focused on the use of robust, manually defined landmarks. Other approaches use biomechanical models deriving tissue properties from the literature, or from image sequences (4D motion analysis). Popular simulation algorithms are Finite Element Methods (FEM), which provide a well-established framework for modeling the mechanical behavior of soft tissues under various conditions and offer high accuracy, but are either not real-time capable or require huge computational resources. Recently, machine learning models (e.g. convolution networks) enable fast, real-time simulations but depend heavily on training data quality and cannot guarantee physical correctness. Hybrid models that integrate machine learning with physics-based priors promise realistic and efficient soft tissue simulation.

Obtaining material properties for simulating soft tissue deformation remains extremely challenging. Tissue models from cadavers and ex-vivo tissue can be obtained [31], but their material properties can be grossly different from those of perfused live tissues. In some cases, live animal experiments may provide some insight, but there are many issues involved, not the least ethical ones. Non-destructive image-based techniques, mainly elastography methods have emerged over the years, using MRI and ultrasound [32].

Ultrasound elastography has advanced significantly [33] and several commercial ultrasound imaging systems now boast elastography options. Nevertheless, ultrasound elastography continues to have major limitations, including sensitivity to patient movement and artifacts at tissue interfaces. Tissue anisotropy and heterogeneity can affect accuracy, and deeper tissues are harder to assess due to wave attenuation.

Magnetic resonance elastography (MRE) is a noninvasive technique for mapping the viscoelastic properties of soft tissues in various MRI-based diagnostic applications [34]. It measures mechanical parameters such as harmonic shear strain, storage and loss moduli, shear wave speed, wave attenuation, and loss angle. In vivo MRE produces parameter maps with spatial resolutions from millimeters to sub-millimeters and dynamic ranges from tens of hertz in patients to kilohertz in small animals. Incorporating MRE into clinical imaging adds only a few minutes to scan time and can be combined with other quantitative MRI methods. However, MRE remains costly, has limited availability, and poses challenges in patient throughput. Current image-based elastography measurement techniques lack in terms of quantitative validity, and calibration and standardization issues further limit their current reliability, especially for complex tissues [35].

The simulation of haptic properties of soft tissue focusses on XR simulators for training stitching, needle insertion, palpation, and procedures using robotic arms [36–38]. A number of approaches for real-time haptic feedback of soft tissue exist that can be distinguished by their mechanical attributes (elasticity, viscosity, and plasticity) and different force propagation models [39, 40]. Fast models for computing linear elasticity exist, but are limited to the simulation of small deformations [41–43] and fracture-based mechanical models [44] were described. A next class of soft issue haptic rendering models are FEM-based models [45], which are optimized for haptic real-time computation, e.g. the precomputation based FEM [46, 47]. While the advancements in haptic simulation of soft tissues have significantly improved the realism and effectiveness of training simulators, challenges remain in achieving perfect fidelity.

### Future Research

The future of deformable registration and tissue modeling lies in the seamless integration of advanced image-based registration techniques with sophisticated biomechanical simulations. The vision is to evolve from basic image-based registration to the development of AI-parametrized deformation models that can accurately simulate the behavior of organs and surrounding structures. Ultimately, the goal is to create Digital Twins that can mimic actual patient behavior in real-time, leveraging multimodal parameter input. This will revolutionize tracking, navigation, and training in medical procedures, enhancing both precision and safety.

To realize this vision, the research community will need to focus on several key areas. First, there is a need to develop fast and accurate 3D models from multi-modal images, ensuring that these models are robust and reliable. Second, automating the registration of 4D data on real tissue during endoscopy and open surgery will be crucial for providing precise and reliable tracking. Third, advancing geometric and haptic simulations of soft tissue will enable robust tracking of instruments, detect potential harm to critical structures, and support robotic surgeries and training simulations. Fourth, establishing a comprehensive database of material properties for various soft tissues across a range of clinically relevant disease states and pathologies will serve as the foundation for highly accurate simulations. Finally, striving for real-time capability will ensure that models and simulations can provide immediate feedback and guidance during medical procedures and their training.

## Interaction and AI

Visualization and simulation in XR environments always require some kind of interaction techniques. The interaction techniques must provide precise control of the information displayed, while at the same time not distracting from the primary surgical task. Combining AI with interaction techniques using XR technologies has the potential to improve situational awareness, reduce cognitive load on the surgical team, as well as maintain focus on the surgical site. This section discusses innovative interaction techniques combined with AI support.

### Interaction Techniques and Technologies

#### Motivation

To unfold the potential of XR in the OR, effective interaction techniques with XR during surgery are crucial. While access to 3D models of patient image data during surgery provides many advantages, the interaction with these XR systems to date is still cumbersome, as the operating room presents unique challenges for interaction. These include a sterile environment, limited and crowded space, and high noise levels. The primary focus of surgeons is performing the surgery, meaning that any interaction with devices is secondary. Efficient hand-eye coordination is crucial, and the focus of attention should ideally be kept at or close to the situs without being in the way. Additionally, the operating room environment is typically stressful, contributing to a substantial cognitive load. Interactions with XR devices should not exacerbate this cognitive load but naturally support and enhance the workflow of surgeons and OR staff. Challenges of XR interaction in the OR that need to be addressed include missing established interaction standards and devices, the usability and comfort of XR devices, suitable interaction techniques for secondary tasks that prevent interruptions, and dealing with the information overload, and a missing integration of scalable systems for all phases of surgery and different user roles.

#### State-of-the-Art

As human-computer interaction (HCI) has moved towards ubiquitous computing, XR, and embodied and multimodal interaction, different interaction techniques have been developed that yield potential for application with XR in the OR. Many have already been explored in research prototypes, such as mid-air hand gestures [48], foot interaction [49], voice interaction [50], and eye gaze [51], as well as combinations of these [52–54]. Especially hands-free interactions [55] and multimodal interactions [56] have many advantages to support tasks during surgery. Matinfar, et al. [57] found that converting position and orientation data into audio enabled users to use hearing for surgical navigation of pedicle screws. Others have investigated presenting information to the tongue for surgical navigation or providing directional or abstract information [58–61]. XR research prototypes have also investigated the use of 3D printed prototypes of visceral organs for interactive surgery planning [8]. There has also been a variety of commercial work to help physicians navigate medical devices into patients in image-guided procedures [62–65].

#### Future Research

##### Interaction Standards and Devices

Each manufacturer has implemented their own solution, so many parallel systems exist with different interaction techniques. Thus, users need to learn and remember each interaction. Standards are needed to simplify this situation.

##### Usability and Comfort of XR Devices

**Fig. 2:**
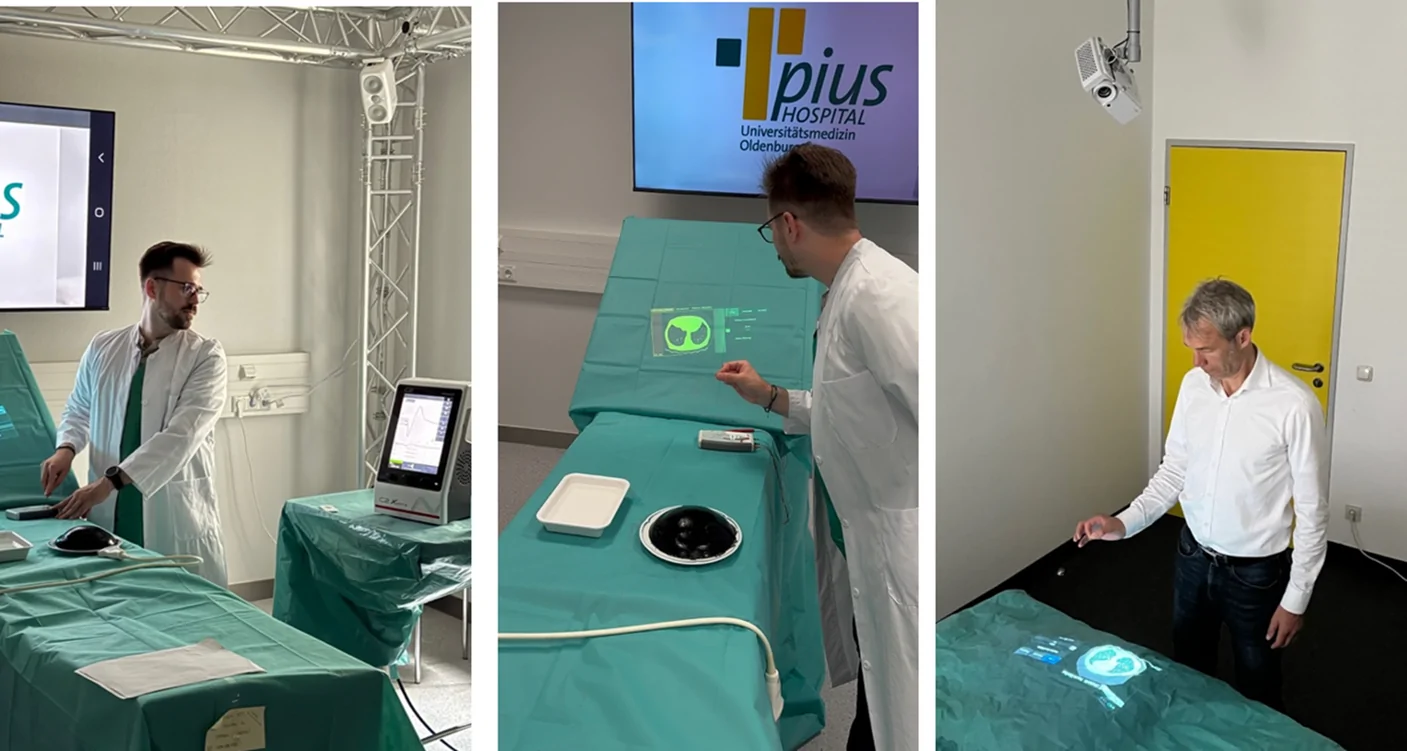
Novel interaction concepts. Left: Surgeons often get distracted by having to look at visualizations of important information placed outside the surgeon’s peripersonal range. Middle: Bringing visualizations close to the operating site, and allowing for gesture-based interaction directly by the surgeon. Right: Information can be visualized without distortions even on non-planar surfaces

HMDs are typically heavy and uncomfortable, especially when used for extended periods [66]. So smaller and more lightweight devices or projections are desirable (for an example see Fig. 2).

##### Suitable Interaction Techniques for Secondary Tasks That Prevent Interruptions

Surgeons need to focus on the operation as their primary task, so any XR interactions should smoothly integrate into the surgical environment. Designing XR interfaces that provide clear, intuitive, and uncluttered information without sensory overload is essential [67, 68]. Techniques such as eye-tracking or interaction with medical instruments could reduce distractions and support the focus on the patient.

### AI and Uber-Agent Interaction

#### Motivation

##### Intelligent Monitoring/Situational Awareness

Surgery involves shifting patient conditions, diverse tasks, and complex team interactions. Since each team member focuses on a specific role, no one maintains full awareness of the entire OR. Humans also struggle with repetitive monitoring, especially during long, stable-seeming procedures. Sustaining attention is particularly challenging when observation must span individuals, teams, and zones. Complications often emerge through subtle cues — minor changes in vitals or behavior that seem benign alone but signal risk in context. An Uber Agent (UA) could analyze real-time and historical data to alert staff when intervention is needed. Fatigue further reduces focus during extended operations. A UA enhanced with AI and XR could track gaze, posture, and cognitive state to detect fatigue or distraction, prompting timely refocus. By combining AI, XR, and multimodal sensing, a UA could enhance human expertise and situational awareness. Effective systems leverage the strengths of both humans and AI [69, 70].

##### Oversight and Context-aware Interactions

 In the OR, timely access to critical information like lab results or patient history and timely assistance when needed is vital for safety and efficiency. An ideal interaction paradigm would enable context-aware interactions, ensuring that relevant information or assistance is provided precisely when needed. The system would leverage its understanding of the ongoing procedure, the user’s position within the OR, and their gaze or gestures to anticipate intent.

##### Coordination of Overall Plans and Changes

A UA could monitor when someone changes or removes a display device and adapt accordingly. If a surgeon removes an HMD and switches to a loupe, the UA should recognize this and adjust how information is delivered. It might prioritize only essential messages and switch to auditory cues to ensure critical information still reaches the intended recipient. Additionally, a critical function of any XR + AI support system is the ability to respond instantly and adaptively during emergencies. In such moments, ongoing processes may need to halt and XR visuals may need to vanish. The da Vinci Surgical System exemplifies this principle: it retracts instruments within seconds during emergencies, enabling unobstructed intervention.

##### Managing Communications, Interruptions, and Distractions

Distractions during surgery are linked to team inefficiency and a higher risk of medical errors [71, 72]. One cause is the widespread use of global displays and alarms in the OR, which are designed for everyone to see and hear. While this ensures that critical alerts aren’t missed, it often results in unnecessary interruptions for individuals not directly involved. A more effective strategy is targeted communication, which reduces disruptions for others. Enabling this approach requires real-time awareness of team member locations and device usage.

#### State-of-the-Art

There is a substantial body of work on use of AI techniques in simulation-based surgical training systems. This use of AI to teach surgery has been enabled by the fact that surgical simulation systems are developed using XR techniques and thus the AI system can leverage rich information concerning the sequence of actions, how they are executed, and their outcomes. Furthermore, the results of AI-based analyses can be effectively communicated back to the student though the XR interface. Task-specific data can be used for structured feedback, and some systems now address more abstract components of surgical performance, such as decision-making under uncertainty [73]. HMDs can provide eye tracking data that can be used to infer focus of attention and even cognitive load, which can be analyzed in relation to user actions and stages of the procedure [4, 74–76]. Despite the successful application in training systems, there has been relatively little application of AI techniques to assist with surgical procedures, largely because of the lack of intraoperative information such systems could make use of. This is now changing with the emergence of XR applications in the OR.

XR systems in the OR enable continuous observation and recording of surgical activity, allowing for the capture of structured, multi-sensor data on instrument motion, field dynamics, and procedural timing. These data have been used to develop AI models that recognize surgical phases, track instruments, and identify anatomical features [77, 78]. In turn, AI-generated outputs can be integrated into XR systems to enhance visual guidance and interface behavior. The integration of XR and AI in the OR is becoming increasingly feasible, with structured data flows enabling real-time adaptation to procedural context [79].

XR has been applied in procedures involving rigid anatomy, such as orthopedics, where spatial alignment between virtual content and physical structures can be maintained with greater consistency [80]. In soft-tissue operations, however, ongoing deformation and changing camera angles introduce variation that current systems still struggle to manage [81]. To address these challenges, AI techniques have been integrated into XR systems to support adaptation to tissue deformation and anatomical changes during soft-tissue surgery, with applications including tissue tracking, landmark recognition, phase identification, and recalibration of augmented views to maintain alignment with the surgical field [82, 83].

AI techniques have been extensively applied to processing of preoperative images, which are used to inform and guide surgical procedures in the OR. AI-driven analysis of medical imaging [84, 85] enables precise mapping of a patient’s anatomy, allowing surgeons to plan procedures with greater accuracy. Prototype systems have explored dynamic overlays, anatomy-aware guidance, phase-specific annotations, and alignment with preoperative imaging during surgery [81]. Some use AI to support overlay of 3D reconstructions based on patient-specific scans, aiding interpretation of anatomy in real time [86, 87], such as blood vessels and tumors [88, 89]. These systems help identify dissection planes and reduce the risk of injury to surrounding structures.

AI-integrated XR systems are changing how information is presented during procedures. These displays can automatically adapt to the instruments in use, the current task, or proximity to critical anatomy. Interfaces that prioritize relevant cues and reduce visual clutter may help limit attention shifts and the need for manual adjustments. For instance, the SurgeonAssist-Net framework uses head-mounted displays to provide context-aware, phase-specific augmented reality guidance, which supports real-time decisions and reduces cognitive load on the surgical team [90]. Yet few systems are designed to interpret team dynamics or guide collective responses during surgery [91]. It is still unclear how surgical teams interpret and respond to AI-assisted prompts in real time, highlighting the need for further study, particularly during critical phases or handoffs [92].

The integration of additional data streams, including audio, sensor inputs, and operative text, may support more complete models of surgical context. In this setting, large language models could assist in interpreting team communication, generating case summaries, or adapting system behavior during real or simulated procedures [79, 93, 94]. Chen et al. [95] described a multimodal large language model designed to process visual and spoken inputs during surgery, with mechanisms for aligning outputs to ongoing intraoperative events.

#### Future Research

##### Intraoperative AI Applications

Broader intraoperative use of AI will depend on consistent model performance across clinical settings and compatibility with existing imaging and data infrastructure. Processing continuous inputs such as video, language, and sensor data in real time remains technically challenging [91, 96]. In addition, successful implementation depends on alignment with surgical routines. Surgeons need to understand and trust the outputs, and interfaces must be intuitive and unobtrusive [97]. The use of intraoperative video and audio data may also raise concerns about privacy, data handling, and how these systems are monitored and used responsibly [94].

##### The UA as a Hidden, Intelligent Collaborator

**Fig. 3:**
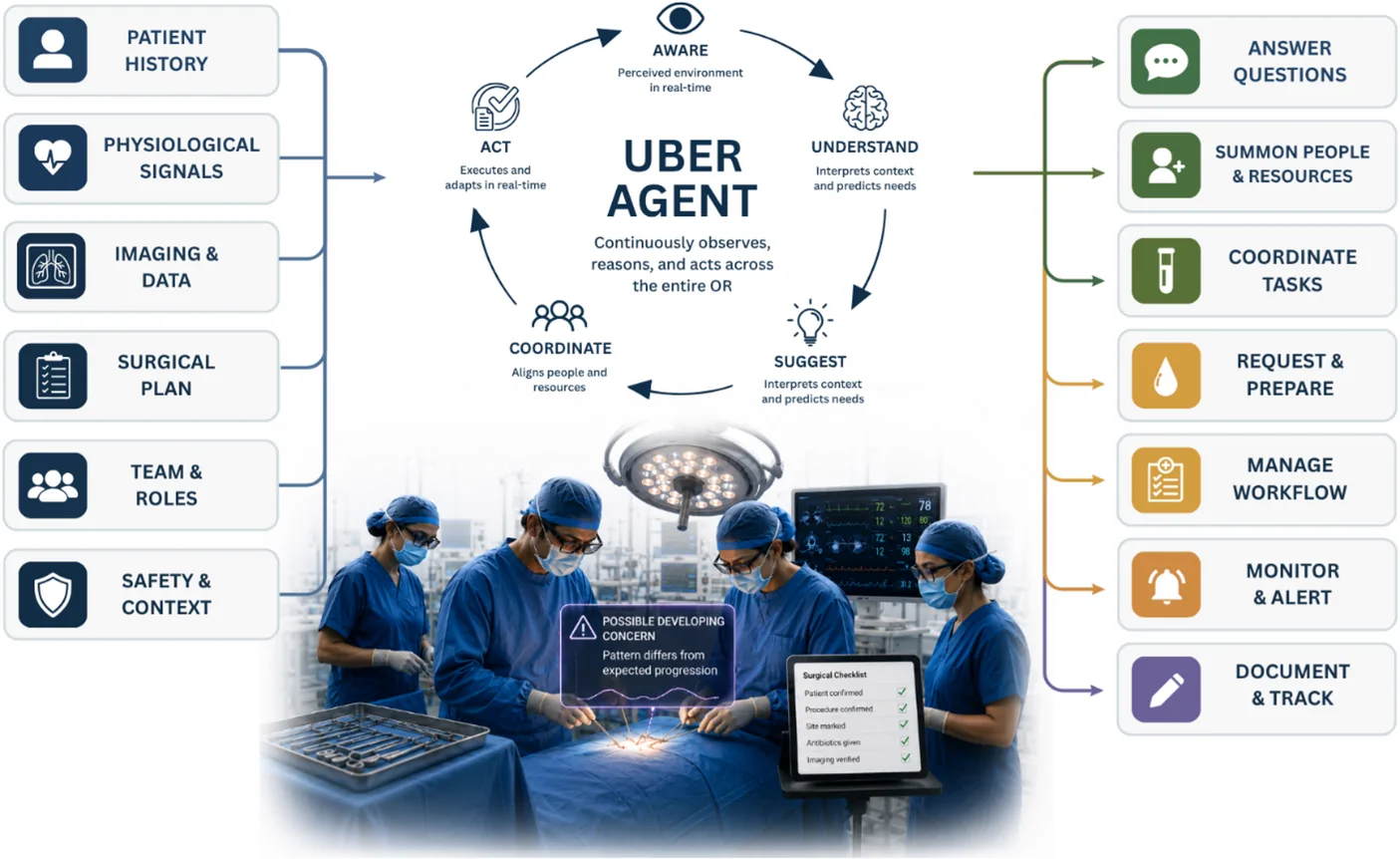
Schematic of the integration of a potential Uber Agent into the procedural workflow in the OR

The UA could function as a hyper-observant, ultra-knowledgeable hidden collaborator, designed to assist, not control (see Fig. 3). One of its core roles would be intelligently directing alerts, messages, and notifications only to relevant individuals or zones in the OR. This targeted filtering would reduce distractions, limit information overload, and combat alarm fatigue—ensuring each team member receives only the information pertinent to their role. The UA could reduce external interruptions by deciding how to handle incoming communications based on who actually needs to be notified, suppressing unnecessary alerts for others. If a disruption does occur, the UA could support resumption by offering subtle, context-aware reminders.

##### Hierarchical, Multi-Resolution Operation

The UA would analyze information at varying levels of granularity based on the surgical team’s structure. It could assess the OR at a high level to detect critical, team-wide events; monitor interactions within specific zones, such as between an anesthesiologist and a surgical nurse; and adjust its focus to the individual level to tailor notifications to each team member.

## Remote Collaboration

Many countries suffer from a concentration of surgical expertise in a few main urban centers. In addition, anticipated substantial demographic shifts are expected to lead to a shortage of skilled medical personnel. Innovative use of XR technologies could facilitate the transfer of centralized medical expertise to distant locations, enhancing the quality of care in underserved areas and during critical situations. This section looks at methods to enable remote medical professionals to provide assistance using virtual reality technologies, the key research challenges that need to be addressed, and the ethical and socio-economic aspects.

### Motivation

Over the past decade, demographic and political factors have contributed to a pronounced shortage of skilled medical personnel. A big factor is a demographic shift, resulting in a reduced pool of health care professionals while the number of people in need for health care is increasing. Furthermore, health care systems in many countries are increasingly oriented towards optimizing efficiency, which often involves the centralization of highly specialized, high-performance care with fewer, more strategically located clinics.

As a result, the quality of healthcare is generally enhanced; however, its accessibility across a nation’s regions typically diminishes. For instance, rural areas are experiencing declining population densities, rendering it infeasible to sustain comprehensive healthcare services. Other regions face a severe deficiency in modern, technology-driven medical care due to economic constraints. This trend is further compounded by the ongoing specialization and advancements in medical diagnostics.

Advances in diverse technologies, including XR technologies, will enable the transfer of centralized expertise to distant locations, even during surgical interventions. This capability is particularly critical in scenarios involving rare diseases, treatments requiring highly specialized knowledge, as well as in time-sensitive contexts, such as triage and initial diagnosis of patients during ambulance transport and in emergencies. Looking further ahead, even in human spaceflight, remote support could provide essential medical expertise in urgent situations.

Today’s remote support is mainly covered via phone (with a few expectations^4^), which lacks highly relevant visuals to enable efficient communication. XR could provide a much richer communication, since it can convey visual details, and it allows for the whole spectrum of communication cues.

### State-of-the-Art

In the past few years, research into remote collaboration has been increasing. Lee and Yao [98] have considered a number of different application settings. Others looked into spatial awareness among collaborating colleagues [99], or the impact of avatar fidelity including audio aspects [100, 101],. The review by Wang et al. [102] provides a good overview on XR remote collaboration involving physical tasks. Garavand & Aslani [103] conclude in their scoping review, that—for now—remote collaboration mostly helps with accessing imaging data.

As robot-assisted surgery (cf. section “Robotics”) is becoming common now, this provides the opportunity for remote telesurgery even without advanced XR systems. Barba et al. [104] provides an overview on what has been achieved so far in this domain; they also point out the well-known remaining challenges like latency and throughput. This is, in particular, a crucial challenge for immersive remote collaboration, where streaming of 3D content directly into VR/AR headsets is done using built-in depth cameras and point clouds [105, 106].

### Future Research

We envision remote collaboration with two slightly different manifestations. In one scenario, the remote medical professional only provides assistance and expert feedback by being virtually present at the scene with full overview of the circumstances and can use the full spectrum of verbal and nonverbal communication cues. Further relevant features like annotations and references are also available. In the other scenario, the remote expert, a surgeon or specialist nurse, can “work-from-home” by controlling a robot acting as their proxy at the medical facility. See the section on Robotics for a more comprehensive overview of the challenges and visions for robotics in XR supported surgery. Either form of remote engagement with the on-site-staff and the patient could lead to remote-assistance centers, where experienced doctors or experts for special or rare cases support medical cases all over the country, or even globally, thereby potentially increasing the quality of patient care.

On the way to successful remote collaboration, we need to overcome three major challenges.

#### Agency and Teamwork

It is important for both the on-site and the remote professionals to have equal conditions and equal agency, to the extent possible possible. The feeling of “being there”, i.e., the place illusion [107], is a contributing factor to feeling like a team which can affect the outcome [108, 109]. To facilitate these aspects we need to research how to make use of the technology to create a strong sense of agency and teamwork, as well as whether training techniques can contribute to this.

#### Standardizing and Integrating Surgical Environments for Seamless 3d Interaction

The setups for remote collaboration need to be as general as possible, so as to allow for flexible usage for all kinds of procedures and locations. This requires methods and technical protocols to connect heterogeneous operating rooms and VR systems. Occlusions of all types of sensor data, which are inevitable in tight spaces, need to be compensated for to avoid confusing artefacts. Also, as space is generally limited, and especially for surgeries on small or delicate structures of the body, we need to investigate the optimal inclusion of remote experts at the local site and how different placements potentially impact the outcome of the procedure. Additionally, we need to do more research into capturing and presenting high resolution, multi-modal information, in particular the visual and haptic senses.

#### Networking and Communication

For transmitting data a standard protocol is needed that ensures that all relevant data can be efficiently streamed to the remote expert’s location, even under changing bandwidth and other quality-of-service during the session.

### Ethical and Socio-Economic Considerations

When talking about remote assistance in a potentially global network of medical professionals, we need to highlight at least a few of the ethical and socio-economic considerations that should be considered. First of all, with more centralized medical centers performing remote health care, we significantly increase the dependency on technology, which further comprises aspects such as reliability (of technology and internet connection), affordability, and availability, especially in countries with different economic situations. Also, such technologies should not exacerbate the lack of access to high-quality health care for the poorer patients in society, but instead provide better health care for all, regardless of individuals’ financial resources. Additionally, we have to consider licensing and certification across borders, as, for example, getting a medical license approved in another country (e.g., Germany) might not be easy due to standards that either country has for medical practice. Countries need to work together to provide the legal framework while keeping patient safety as a priority. Lastly, if surgeons eventually specialize in and perform only one single procedure remotely all over the world, this might produce “single skill experts” who might miss the bigger picture or who get bored.

## Robotics

Robotic surgery offers enhanced precision and minimal invasiveness, leading to faster recovery times and minimized risk of complications. The integration of XR technologies such as haptic interfaces and AR overlays offer the potential to increase usability and support remote control, simultaneous operation of multiple robots, and real time tracking and registration. This section looks at the role of XR in enhancing the capabilities of robotic surgery, as well the challenges involved.

### Motivation

Robotic surgery (RS), also known as robot-assisted surgery, is a minimally invasive technique where robots are used to perform surgery. They offer enhanced precision, control, and flexibility, resulting in smaller incisions, reduced blood loss, faster recovery times, shorter hospital stays, minimized risk of complications, and reduced surgeon fatigue [110, 111]. RS can also enable manipulation of structures that are too small to be perceived and manipulated by non-augmented humans [112, 113]. However, RS requires specialized training and incurs higher costs.

A typical RS system consists of robotic arms equipped with surgical instruments and a high-definition (HD) camera that provides a 3D view of the operating space. However, the field of view is often limited, as procedures are performed through a narrow opening, restricting the surgeon’s broader perspective. AR can enhance the visual field by overlaying preoperative imaging/plans onto the operating space. XR assistants could offer an efficient way to interact with RS systems without requiring direct tactile manipulation, enabling the use of multiple robotic assistants simultaneously and reducing the need for human experts. XR could also enable remote surgery, further addressing resource shortages. This would involve the remote surgeon operating on a virtual model of the patient using a haptic enabled RS system with their actions precisely mirrored on the patient through a connected RS system.

### State-of-the-Art

RS has achieved notable clinical successes, including the first fully robotic double lung transplant [114] and dual-robotic prostate removal surgery [115]. Several RS systems are currently used in clinical practice. The choice of system depends on factors such as the surgical application, surgeon’s expertise, and available resources. Intuitive Surgical’s da Vinci Surgical System [116] is the most widely adopted RS system. It features wristed instruments that mimic hand movements but with a greater range of motion, and provides a magnified, HD 3D view of the surgical area. Medtronic’s Hugo Robotic-Assisted Surgery system [117] has emerged as a noteworthy competitor and offers modular arms, an open console with 3D glasses, and compatibility with standard laparoscopic instruments. The Versius system by CMR Surgical [118] features independent, modular arms that allow for quick and easy setup and portable bedside units that adapt to various operating room layouts. Other RS platforms include Stryker’s Mako System for orthopedic surgery, Brainlab’s Cirq system for spine and cranial procedures, and the Symani Surgical System by Medical Microinstruments for microsurgery.

The integration of cutting-edge technologies into RS systems is enhancing their capabilities and improving patient outcomes. AI is increasingly being used to provide intraoperative assistance [119, 120]; Research is underway to develop autonomous RS systems that can perform specific surgical tasks without direct human intervention [121, 122]; Haptic interfaces that provide more intuitive control over robotic instruments are being explored [123].

### Future Research

Future XR enabled RS systems will support autonomous procedures, remote control, and simultaneous operation of multiple robots. While the convergence of XR and RS has the potential to revolutionize surgery, it also introduces challenges that must be addressed to ensure safety, efficacy, and practical implementation.

#### Real-Time Tracking and Registration

While significant work has been conducted in these fields [124, 125], accurate lag-free internal tracking and patient registration that support AR overlays in RS still remain a challenge. Miniaturized, biocompatible sensors integrated into surgical tools will enable high-fidelity positioning without interfering with the procedure; Reliable fusion of multiple sensor modalities (e.g., electromagnetic/optical tracking, inertial measurements, and real-time imaging) will ensure robust and redundant tracking; Reliable and fast registration algorithms should align preoperative data with the patient’s/robot’s intraoperative position, even with limited landmarks or posture changes; Tracking systems and registration algorithms must compensate for tissue deformations.

#### Network Infrastructure and Latency

As technology advances, integrating XR into RS has the potential to drive innovation beyond current frontiers, establishing a new paradigm in surgical practice. However, this will require extremely low latency and high bandwidth to ensure real-time control and feedback [126, 127]. This is a significant hurdle, especially in areas with limited network infrastructure. Research must focus on optimizing bandwidth and leveraging next-generation network technologies; edge computing (data processing closer to the surgical site) could reduce transmission delays; algorithms that predict potential network issues and provide preventative measures will ensure patient safety.

#### 3d Bioprinting

The use of XR to control 3D bioprinting robots in real-time will allow for in-situ tissue creation to repair damaged tissues or create new structures. This poses challenges in developing biocompatible materials and precise deposition techniques.

#### Shape-Changing Robots

Robots that can adapt their shape to navigate anatomical structures in complex and constrained spaces will minimize tissue damage and incision size. This introduces challenges in developing biocompatible, controllable, shape-changing robots, as well as in creating intuitive ways to manipulate them.

#### Swarm Robotics

Coordinating swarms of miniature surgical robots through an XR interface enables multiple robots to collaborate on specialized tasks. This requires methods to autonomously or semi-autonomously control large robot swarms, clearly present their state and actions to the surgeon, and ensure precise localization and safe operation within the body.

## Surgeon Perspectives

XR technology holds great potential to transform surgeons’ daily work throughout their careers—whether in acquiring and maintaining skills, sharing expertise across great distances, or supporting critical steps during surgery. Through digital simulations, connected mentoring, and integrated intraoperative visualization, XR could reduce the significant costs and ethical burdens of traditional training and surgical practice, bring expert knowledge to underserved regions, and alleviate the cognitive strain that contributes to fatigue and burnout. This section discusses how XR can support surgeons before, during, and after individual procedures.

### Motivation

Acquiring and maintaining surgical skills is resource-intensive. Established frameworks for surgical training emphasize targeted, repeated practice in safe environments before operating on patients [128, 129]; however, the ability to provide such training opportunities is limited by cost and availability. Highly realistic physical phantoms incur recurring costs for consumables across an entire training cohort, while animal and cadaver models entail additional costs for procurement, facilities, and ethical oversight and often differ in their material properties from perfused living tissue (see Sect. 3) [31]. This ethical dimension is enshrined in the 3R principle and in EU legislation on the use of animals for scientific purposes, both of which explicitly promote validated animal-free alternatives such as simulation [130, 131].

Proctoring—the direct supervision of a surgeon by an experienced colleague during early cases with a new technique or device—is a cornerstone of safe skill transfer and is embedded in the credentialing and privileging guidance of major professional bodies [132]. However, proctors are scarce, travel is costly, and scheduling is difficult, which can delay the safe adoption of beneficial techniques. This is amplified in remote and underserved regions, where surgical expertise is concentrated in urban centers (see Sect. 5) and where trainees may have little access to either mentors or physical training resources.

Intraoperatively, a surgeon must integrate fragmented data streams and maintain awareness of critical anatomy under limited-view conditions. These demands can induce high cognitive load and lead to inadvertent injury. This is particularly relevant to minimally invasive and endoscopic surgery, where a narrow 2D or stereoscopic 3D view constrains spatial understanding (see Sect. 6).

Communication among the members of the surgical team is an essential aspect of surgery. Currently, the primary form of communication is verbal. For instance, controls for lighting, CO₂ insufflation, operation of X-ray fluoroscopy units, or specialized medical devices such as neurostimulators must be communicated verbally from the sterile surgical team to a non-sterile team member. In the acoustically demanding environment of the operating room, communication misunderstandings can arise, disrupting the surgical workflow. This leads to increased workload for the responsible surgeons and represents a potential source of error, thereby compromising patient safety [133]. Introducing technologies that enable members of the sterile surgical team to directly control devices, as well as intelligent information displays that anticipate information needs could significantly reduce the risks associated with verbal communication.

Finally, surgery is associated with long hours, high cognitive load, and a well-documented tension between professional obligations and family life; work–home conflict is strongly associated with surgeon burnout [134, 135], which remains prevalent across training and practice [136, 137]. Reducing the time, travel, and cognitive burden of training, mentoring, and operating could therefore benefit surgeon well-being as well as patient safety.

### State of the Art

XR-based simulators have demonstrated measurable improvements in surgical skill, confidence, speed, and accuracy compared with traditional methods, and can substantially reduce reliance on consumable phantoms and animal models [4–7, 138]. Scoping and systematic reviews confirm rapid expansion of XR in medical education and transfer of simulator-acquired skills to intraoperative performance [5, 7], while haptic-enabled simulators increasingly reproduce force feedback for procedures such as total hip arthroplasty and minimally invasive endodontics, now supported by randomized controlled trials [6, 138]. Because these simulators are reusable, they offer a favorable long-term cost profile relative to per-use physical models.

For mentoring, XR extends proctoring beyond physical co-location. Remote and telementoring platforms allow an expert to annotate the operative field and guide a less experienced surgeon in real time [98, 102, 103], and surgical societies have begun to formalize telementoring as a valid supplement to on-site supervision, provided that connectivity, accountability, and patient-safety requirements are met [139, 140]. As robot-assisted surgery becomes routine, telesurgical procedures on phantoms have been demonstrated over distances exceeding 300 km using 5G networks [104, 127], illustrating how expertise might reach remote regions without requiring the proctor to travel (see Sect. 5).

Within the operative field itself, information fusion and risk-structure alerting are advancing. AR systems have integrated ultrasound and endoscopic feeds into head-mounted displays to reduce attention shifts [11, 12], and commercial platforms already fuse microscope, ultrasound, and reconstructed imaging into the workflow [13]. AI-assisted overlays of patient-specific 3D reconstructions can highlight blood vessels and tumors, help identify dissection planes, and thereby reduce the risk of injury to surrounding structures [88, 89], while soft-tissue simulation can, in principle, alert the team when a critical structure is endangered (see Sect. 3). In endoscopic surgery, stereoscopic 3D visualization and, increasingly, multimodal large language models designed to interpret intraoperative video and speech point toward richer contextual guidance [93, 95]. Nonetheless, most of these systems remain confined to research settings, lack standardization, and have limited validation of long-term skill retention, cross-border credentialing, and intraoperative reliability (see Sects. 5 and 9) [141, 142].

### Future Research

Future work should aim to make high-fidelity, low-cost surgical training and expert guidance ubiquitously available while reducing the burden on the individual surgeon. Priorities include developing validated haptic tissue models—supported by comprehensive libraries of material properties (see Sect. 3)—that can replace animal and cadaveric models for a wider range of procedures; and establishing standardized, interoperable telementoring and telesurgery protocols that address latency, licensing, and liability across regions and national borders (see Sects. 5 and 6).

Topics that research to support surgeons intraoperatively should address include information fusion, deformation-aware overlays, alerts to potential dangers, and sterile-field control of devices. As discussed in Sects. 2 and 3, robust information fusion is needed so that preoperative imaging, live endoscopic 3D video, and sensor data are perceptually aligned in a single field. For endoscopic and robot-assisted surgery, integrating deformation-aware overlays and stereoscopic depth cues could compensate for the restricted field of view. Reliable, alerts to potential dangers during the operation, e.g. low-latency proximity alerts that warn when instruments approach risk structures—while explicitly conveying tracking uncertainty (see Sect. 9)—will be essential to earn surgeons’ trust. Sterile-field control via contactless HCI could not only enable more natural interaction but also restore full control over human-machine interaction to the responsible surgical team within the sterile field [56]. Biometric methods such as eye tracking, gesture control and even thought control could represent future solutions, in combination.

Equally important is research into how these technologies affect surgeon workload and well-being. Reusable simulators and remote proctoring could reduce travel and enable more flexible “work-from-home” mentoring and, eventually, remote operating, potentially improving work–family balance and mitigating burnout. Yet the same connectivity risks blurring professional and personal boundaries, and the emergence of highly specialized “single-skill” remote experts raises concerns about professional satisfaction and breadth of competence (see Sect. 5). Rigorous, multi-dimensional evaluation—capturing skill transfer, cost-effectiveness, cognitive load, ethical acceptability, and quality of life—will be essential to ensure that XR genuinely benefits surgeons as well as patients (see Sect. 10).

## Patient Perspectives

Not only in the operating rooms, but also before and after the operation, novel XR technologies bear great potential to improve the patient’s experience by reducing anxiety, improving communication, and empowering patients to be active participants in their healthcare decisions. Through immersive visualization, XR can provide personalized immersive and understandable explanations of procedures and their various possible outcomes. This section discusses how XR technology can provide support to improve the patient experience before, during, and after surgery.

### Motivation

While the use of XR in the operating room has traditionally centered around enhancing the capabilities of surgeons and medical staff, there is growing interest in exploring how these technologies can also transform the patient’s experience, in particular improving the decision-making processes and reducing preoperative anxiety, by providing a more immersive and transparent understanding of the procedures. This shift highlights the importance of empowering patients as active participants in their health care.

VR for distraction and relaxation has shown promising results in reducing patient anxiety and pain in surgical settings. VR and AR also bear great promise for patients to participate in preoperative planning. The interactive experience fosters better communication between patients and healthcare providers, giving patients the chance to ask questions and gain a deeper understanding of the procedure, ultimately improving shared decision-making.

A particular challenge when designing XR systems to successfully engage patients are usability and accessibility. The technologies must be intuitive and comfortable for patients of all ages, abilities, and health conditions. Other challenges are, of course, cost and integration, which can present significant barriers to the widespread adoption of XR technologies in perioperative situations involving patients.

### State-of-the-Art

From the patient’s perspective, research on using XR in the operation room has mainly focused on pain and anxiety, and improving communication and compliance. A systematic review and meta-analysis by Kodvavi et al. [143] found that use of VR led to a significant reduction in post-procedural anxiety, as well as both intra- and post-procedural pain in adults. In the pediatric population, VR has primarily been explored as a distraction technique. Eijlers et al. [144] and Addab et al. [145] conducted a systematic review and meta-analysis in pediatrics, revealing large effect sizes for VR as a distraction intervention to reduce pain and anxiety across a variety of medical procedures.

While the evidence for anxiety reduction through VR appears consistent across different patient groups and procedure types, its impact on pain can vary. A systematic review and meta-analysis focusing on patients undergoing cardiac procedures by Micheluzzi et al. [146] found that VR effectively lowered anxiety levels, but, in this specific context, it did not demonstrate a statistically significant difference in pain reduction.

VR and AR show promise in improving patient communication and compliance [147, 148], especially for explaining complex procedures like epilepsy surgery [149]. VR for procedure visualization offers patients a clearer understanding of their upcoming surgery by creating interactive 3D representations of the procedure [150].

Overall, the current state of the art suggests that VR is a valuable tool for improving the patient experience in the periprocedural period. While its application for pain management might be context-dependent, its role in anxiety reduction appears more consistent. The potential for VR to enhance patient communication and compliance by providing immersive and understandable explanations of procedures is also significant. Further research is crucial to fully understand its capabilities and address any associated challenges.

### Future Research

The future of XR in the operating room will increasingly center on personalized, patient-centered experiences designed to enhance decision-making and reduce anxiety. At the same time, healthcare professionals will be empowered to deliver more transparent, empathetic, and data-informed guidance throughout the perioperative journey.

The integration of haptic feedback into VR experiences for patients in the OR could be a promising direction for future research. For instance, synchronized, even personalized haptic feedback could map real-time surgical events—such as injections, incisions, or manipulations—to corresponding tactile sensations in the VR environment. Specific patterns of calming vibration or gentle touches — for instance, a butterfly landing on the arm to signify an injection, or a soft breeze to simulate the flow of oxygen — could be used to modulate pain perception during and after surgery by stimulating nerve fibers, thus dampening pain signals.

The use of VR to provide emotional support during surgical procedures is an emerging area with significant potential to enhance the patient experience. One promising application is the virtual presence of loved ones. By enabling patients to interact with family members and friends in a shared virtual environment—especially during procedures performed under local anesthesia—VR could help reduce anxiety and foster a sense of comfort and connection. XR systems could be designed to respond adaptively to the patient’s emotional and physiological state, by incorporating sensors to monitor heart rate, skin conductance, and breathing patterns. For example, if a patient exhibits signs of heightened stress or discomfort, the system could automatically shift to more calming imagery, soothing sounds, or reassuring interactive elements.

AI-powered embodied virtual assistants integrated into VR environments could offer transformative possibilities for improving patient education, emotional support, and compliance, particularly during the postoperative phase. Such virtual agents could respond to multi-modal patient queries and interactions with conversational fluency, even recognizing nuanced emotions and concerns. By training AI models on extensive medical datasets and individual patient profiles, these systems could deliver responses that are both clinically accurate and in line with a patient’s treatment plan. Adaptive systems could tailor the information, visual presentation, and interaction style according to the patient’s preferences, cognitive capabilities, and emotional state. Furthermore, AI assistants could recognize the patient’s emotions and offer reassurance, guided relaxation techniques, or referrals to support groups, thereby fostering emotional resilience during recovery.

VR environments could also be used for cognitive testing, for instance, during awake brain surgery. This technique offers a unique opportunity to assess and track cognitive performance in real-time, allowing clinicians to continuously monitor critical functions such as language, memory, attention, and executive processing while surgery is underway. At the same time, patients can remain immersed and focused, reducing anxiety and improving compliance with testing protocols during this delicate procedure.

Post-operatively, the use of virtual reality in the recovery room represents a promising frontier for preventing or mitigating post-operative delirium, particularly in older or cognitively vulnerable patients. By delivering immersive, *early cognitive stimulation* during the critical recovery window, VR has the potential to support cognitive reorientation, reduce anxiety, and enhance overall recovery outcomes. *Relaxing VR environments*, such as peaceful nature scenes or guided meditations, can promote calm and reduce anxiety—two factors strongly linked to delirium onset. In addition, VR can support *virtual social interaction*, which could help reduce feelings of isolation, a known contributor to delirium. Finally, VR environments can integrate *reorientation tools*, such as virtual clocks, calendars, and familiar imagery, to reinforce the patient’s sense of time, place, and identity, supporting a smooth cognitive recovery.

VR could also help to prepare mentally for life after surgery by *post-surgical life simulation*, where patients can interact with a virtual environment that mirrors the activities and routines they might encounter after surgery. For example, a patient undergoing joint replacement or neurosurgery could practice using assistive devices, self-care, or interacting in social situations through VR. This preview can reduce uncertainty, set realistic expectations, and empower patients to feel more confident and in control of their recovery journey. In addition, *emotionally preparing for changes*—such as altered body image, speech difficulties, or lifestyle limitations—can be facilitated through carefully designed VR scenarios.

## Robustness and Uncertainty

Since the operating room is a high-stakes work environment, ease of use, reliability, and stability of XR platforms is crucial to their widespread adoption in practice. This section focuses on key challenges to enable XR technology to move beyond research environments into clinical practice, as well as techniques and strategies to enable such adoption.

### Motivation

Operating room time is expensive, and surgeons are under pressure to perform more and increasingly complex procedures while minimizing invasiveness. In this context, XR technology can help to dynamically present only the necessary, timely information relevant to the current surgical phase, effectively streamlining decision-making processes. Moreover, XR offers the potential to convey uncertainties, measurement errors, and deviations from preoperative planning, enabling surgeons to manage errors proactively and intuitively [151].

However, before XR solutions can be reliably integrated into clinical practice and deployed routinely in operating rooms, several significant technical and usability challenges must be addressed. These pose significant obstacles to effective clinical adoption and must be carefully considered if XR is to achieve its full potential in improving surgical outcomes and patient safety.

### State-of-the-Art

**Fig. 4:**
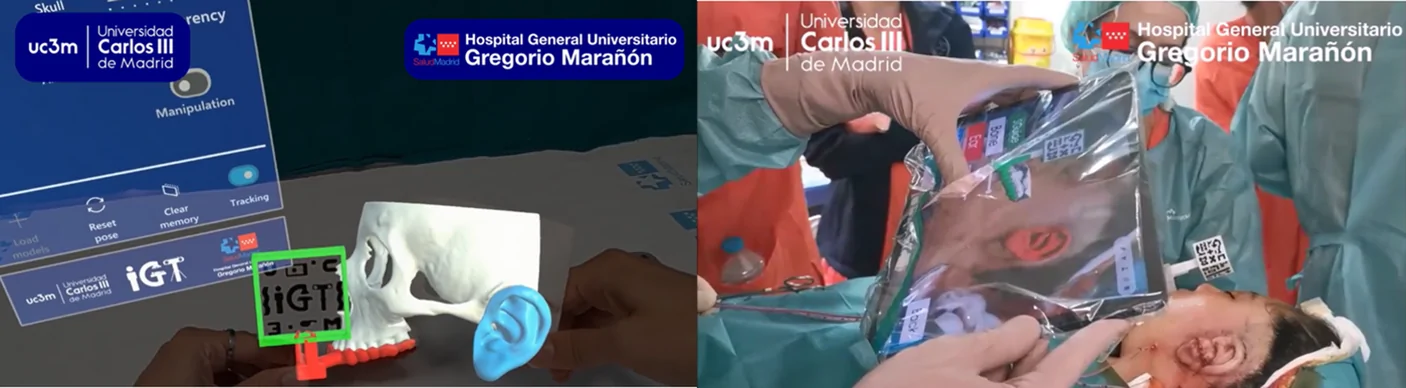
Microtia surgery guided by AR. Left: a 3D‑printed registration pattern attached to the patient’s upper jaw enables visualization of the planned position of a reconstructed ear during surgical simulation [152]. Right: the same solution can be deployed on a tablet device (inserted in a sterile bag) for surgical guidance [153]

The deployment of XR systems remains limited to research environments and isolated case studies. These solutions predominantly rely on head-mounted displays (HMDs), although mobile devices have also been explored to some extent [152–154] (see Fig. 4). Current solutions have only been tested with highly motivated and receptive surgeons who are more willing to tolerate system deficiencies [155], and mainly for training and pre-operative planning [151]. Such favorable research conditions starkly contrast with the complex demands and constraints of actual clinical workflows, where robust performance, reliability, and seamless integration are prerequisites rather than optional attributes.

The clinical adoption of XR in surgery is hindered by multiple critical shortcomings in current-generation systems. Current XR systems typically require cumbersome and lengthy preparations, negatively impacting usability, increasing surgical time, and ultimately limiting acceptance among surgical staff [156–158]. Adoption of these new XR solutions will only be possible if surgeons can reduce operating room time or significantly improve surgical outcomes.

**Fig. 5:**
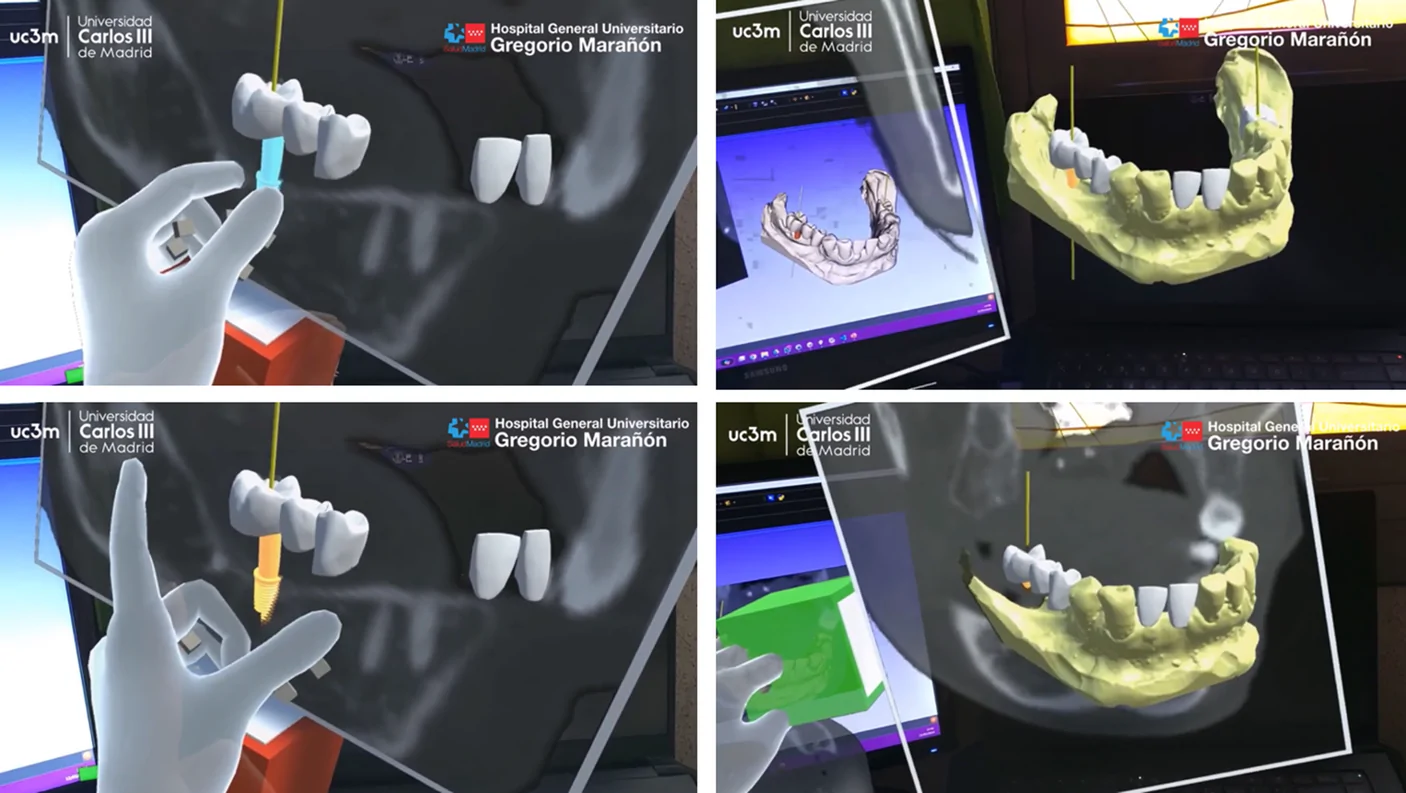
Interaction with medical images using HMDs. The VR visualization is connected to 3Dslicer at runtime [159]. Top left: user places the implant interactively using hand gestures. Bottom left: final position. Top right: simultaneous visualization in 3D slicer. Bottom right: verification of the planned implant position w.r.t. the patient’s real anatomy in 3D slicer

Furthermore, existing XR systems lack robust mechanisms for seamless and fluent integration and transfer of patient-specific data, such as volumetric images, segmentation masks, and surgical planning information. Current platforms often necessitate manual or semi-manual transfer of patient data to XR devices (see Fig. 5) or patient-specific applications [159, 160]. Another critical issue is robustness under actual operating room conditions, which typically involve highly variable illumination conditions, including intense, bright surgical lights and reflective, texture-less surfaces. Such conditions pose severe challenges for AR visualization, registration, and tracking algorithms [161–164]. Current XR devices rely heavily on proprietary algorithms [165], rendering direct measurement or quantification of tracking errors and uncertainties difficult and limiting repeatability. Consequently, the surgeon lacks explicit awareness of the degree of tracking accuracy or possible deviations, severely limiting trustworthiness and clinical applicability.

Although some standardized interfaces exist, such as OpenXR [166], the limited adoption creates compatibility issues between devices from different vendors, making long-term investment in XR technology uncertain for healthcare institutions. Moreover, companies investing in XR face financial and operational risks, as seen with Microsoft’s withdrawal from the XR market [167], leaving HoloLens and other Windows Mixed Reality devices obsolete.

Finally, many research groups currently repurpose consumer-grade XR devices—which are not explicitly designed, tested, or certified for medical use—as integral parts of their surgical navigation systems. Thus, consumer hardware represents an unquantified clinical risk that requires careful evaluation and formal testing under realistic clinical conditions.

In conclusion, despite promising early research, the current state-of-the-art in XR remains significantly limited in robustness, usability, reliability, trustworthiness, and integration readiness. Resolving these key limitations—through improved hardware design, open-standard interfaces, robust tracking algorithms that explicitly measure and communicate uncertainty, streamlined clinical workflows, and clear regulatory pathways—is essential before XR technologies can realize their transformative potential in surgical practice.

### Future Research

Medical device companies typically operate within long innovation cycles, often spanning around ten years. Throughout this period, they are obligated to provide consistent support, maintenance, and regulatory compliance for their devices, ensuring long-term reliability and patient safety. This model clearly contrasts with the rapid innovation cycles in the XR industry, where new headsets and platforms are released annually, and previous generations are frequently deprecated within a few years. This stark difference in product life cycles requires careful strategies to bridge the gap between fast-moving XR innovation and the long-term support expectations of the medical field. What applies for hardware is also valid for software libraries where medical device companies need to establish very long-term software library version management. All these restrictions highlight the importance of greater interoperability and standardization across hardware and software platforms.

The need for a more *sustainable ecosystem* begins with the software platforms, such as Unity [168] or Unreal Engine [169], which inherently bear the risk that the providers may change licensing terms or modify critical components such as the rendering pipeline between versions. Examples of such challenges include Microsoft’s decision to deprecate the Windows Mixed Reality (WMR) framework in 2023. Since it was extensively used by various third-party companies, such as Hewlett-Packard, many XR headsets became unsupported within challenging timeframes for medical device companies. In contrast, open-source platforms, such as the Godot Engine [170], provide greater transparency and control. Future research should prioritize the development of *open*,* universal standards*, such as OpenXR [171], which enables interoperability across devices from different manufacturers.

In order to foster surgeons’ trust, XR glasses must transparently communicate tracking *accuracy and error states* in real-time. Research into reliable, intuitive methods of visualizing the estimated or expected accuracy of the devices or methods currently being used by the surgeon—such as confidence indicators or dynamic visual cues—will be essential to ensure safe, informed decision-making during procedures. Interim solutions involving external tracking cameras highlight accuracy improvements [172]. Further research should investigate hybrid systems combining external high-precision tracking (e.g., using an Invar bar) integrated with XR headsets, eventually transitioning toward fully integrated, robust tracking systems within the XR glasses.

Currently, there is an absence of comprehensive standardized *protocols for evaluating* XR devices as medical equipment. Some authors have proposed initial solutions that could be replicated [163], but future research must define robust benchmarks and realistic mock-up OR scenarios, to rigorously assess XR system reliability, performance, and safety under clinically relevant conditions. (For further discussion, see next section, Evaluation.)

## Evaluation

Given the potentially high impact of using XR technology in the OR, careful evaluation of proposed systems is essential. This must consider multiple dimensions, including direct clinical impact, impact on the patient, as well as financial aspects. Current research focuses primarily on the direct impact of XR systems on the surgeon and the surgical procedure, as well as technical system factors. This section examines the significant challenges in effectively evaluating XR technologies supporting surgery, existing work and approaches for evaluation, and promising research directions to address the numerous open issues.

### Motivation

Every newly developed tool or technology must undergo testing for its usability and potential benefit in a clinical setting. This is especially true for technologies, such as XR, that could change the workflow considerably. Ideally, this is achieved through early, iterative, and user-centered evaluation starting with a comprehensive requirements analysis. In addition, in the context of medical device development, it is particularly important to consider relevant regulatory requirements for evaluation procedures at an early stage.

Evaluating new technologies, especially XR technologies, can be very tricky, for a number of reasons. There are numerous different metrics that could make sense for any new XR tool. In addition, there are complex ethical and regulatory issues if a new tool is to be evaluated prospectively in a real clinical setting; however, if tools are evaluated in a highly controlled setting, then it is difficult to prove the applicability of the results for a real OR. Development of concrete meaningful metrics for use of XR in the OR is still an underexplored area.

### State-of-the-Art

The use of XR in surgery—and consequently the evaluation of human-computer interaction (HCI) in this context—originated in neurosurgery [173]. With the widespread introduction of minimally invasive surgical techniques, HCI has become an indispensable component of surgery. Consequently, the evaluation of such interactions has become a well-established research field. For instance, Grätzel et al. [174] already investigated the use of a contactless mouse in the operating room, and since then contactless input modalities have remained a continuous research focus, naturally driven by the necessity to maintain the sterility of the surgical team throughout procedures [56]. Current state-of-the-art research typically assesses the performance or usability of a tool or XR technology from two distinct perspectives: the first one is the impact on clinical outcomes, which can be considered from both the surgeon’s and the patient’s perspective; the second perspective is the practical usability in daily clinical practice, including financial aspects.

**Fig. 6:**
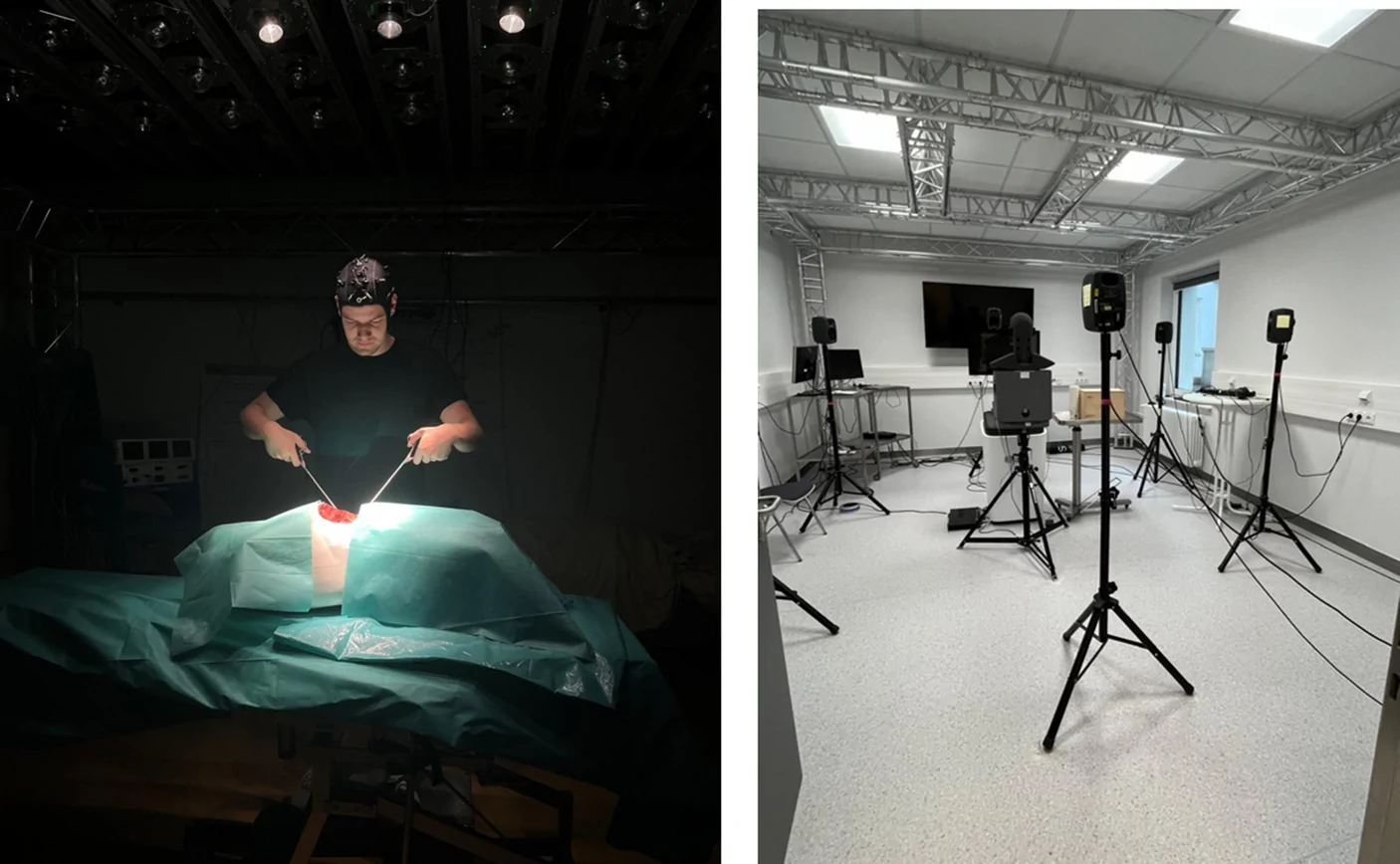
State-of-the-art OR simulations (living labs) allow for evaluation of new technologies using various objective measurements, such as EEGs. Left: A novel lighting concept is evaluated [52]. Right: Acoustic conditions in the OR can be simulated using surround sound systems [182]. Lastly, technical performance and data integration factors such as latency, frame rate, data integration rate, and interoperability index [175, 178], are also crucial in evaluating the effectiveness of an XR system, since they heavily impact usability

Current research clearly focuses on the surgeons’ perspective. Directly and objectively measurable factors commonly used to assess XR tools in the OR include surgical accuracy, procedure time, and error rate [175, 176]. Other factors such as surgeon confidence and cognitive load can be assessed using questionnaires. Commonly implemented ones are the NASA-TLX [12], Surg-TLX, and the System Usability Scale [176–178]. In addition to those subjective measures, bio-signals (e.g., mobile EEG, skin conductance, and electromyography) can provide objective metrics for workload, stress, and fatigue, experienced by surgeons during the use of XR tools (see Fig. 6) [179–181].

### Future Research

Currently, most of the HCI research with regard to XR focuses on training scenarios or only simulates OR conditions and, with the exemption of neurosurgery and vascular surgery, very few studies explore XR systems during actual surgery [182, 183]. This is probably most often due to problems with obtaining ethics approval and/or data security issues. Therefore, up until now there is very little to no data available with regard to patient centered outcomes of HCI evaluation in the OR. From a patient’s perspective, metrics pertaining to how XR technologies improve patient care and recovery are probably the most relevant, and should therefore be included in future evaluation studies. These include, but are not limited to, length of hospital stay, readmission rate, functional recovery score (e.g., Karnofsky Performance Scale, NIH Stroke Scale, or the Short Physical Performance Battery), quality of life (e.g., Short Form 36 Health Survey), and pain scores (e.g. VAS or NAS). If future research includes patient perspective evaluation, it should be kept in mind that it is particularly challenging since it is by nature subjective and patient preferences regarding outcomes may differ significantly before and after experiencing the surgical outcome.

Financial impact metrics like costs, return on investment, and DRG reimbursement can help assess the economic benefits of implementing an XR tool in a surgical setting. However, as with the patient’s perspective, these metrics are currently not considered when evaluating XR-centered HCI in the OR. This appears to be an important research gap that must be addressed in the future, especially given that in healthcare systems heavily focused on economic incentives, the cost of acquiring and deploying such XR systems is a key factor in determining whether they will actually be used.

Finally, for future research, it is worth mentioning that there are also interactions between the various factors. For instance, a system may achieve ease of use by hiding important details, and length of hospital stay or readmission rates influence treatment costs. Accordingly, the selection of an appropriate approach to evaluation requires careful consideration.

## Discussion and Conclusions

The implementation of intelligent XR environments in clinical care represents a significant technological advancement in healthcare delivery. The practical applications of XR technologies have already demonstrated a measurable impact on clinical procedures, such as shorter procedures, reduced errors, and improved surgical outcomes [151, 184, 185]. Our analysis of current capabilities and future developments reveals, however, several critical areas for investigation and development needed to address the major challenges in the modern operating room that motivated this study. Here we distill some of the key takeaways by summarizing those challenges, the ways that XR can potentially help to address them, and the key issues that need to be addressed to realize that potential.

### Information Overload in the or, Resulting in High Cognitive Load and Lack of Situation Awareness

Modern ORs contain a large variety of displays, each providing a different type of information. This often results in high cognitive load, loss of focus on the surgical site, and reduced situation awareness. XR technology can address this problem in a number of ways. This includes integrating a variety of information (e.g. vital signs, pre-operative imaging, endoscopic feeds), facilitating interaction with displayed information, and automating selection of the most relevant information to display at the right time and tailored to the needs of each member of the surgical team. Realizing these capabilities will require solving a number of challenges. We need the ability to determine how to best personalize information while providing members of the team with a common shared understanding of state of the surgical procedure. Personalized information will need to be displayed through an HMD, so that comfort and accuracy of display are crucial. Interaction techniques need to be able to control the information displayed without distracting the user from their central tasks or distracting others in the OR. Thus, techniques such as eye tracking, gesture recognition, and contextualized interpretation become essential. Finally, AI integration will be essential to intelligently select the right information to display at the right time. This includes the ability to recognize the current state of the surgical procedure.

### Need for Accurate and Easily Interpretable Rendering of Pre-Operative Medical Images, Particularly Given the Current Surgical State

Surgeons should ideally be provided with a coherent visual field in which real (surgical site) and virtual (e.g. pre-operative imaging) elements are perceptually aligned in terms of spatial position and semantic relevance. XR technology can make major contributions here by registering pre-operative images with the current state of the patient anatomy as anatomical features dynamically change during surgery. XR could also provide accurate portrayal of depth, lighting, and color when fusing virtual and real images. Realizing this will require significant advances in fast and accurate 3D tissue models from multi-modal images, as well as registration with real tissue during the surgical procedure. This information then needs to be displayed in a way that merges it with the visible surface of the operating field and at the same time provides an understanding of underlying, hidden structures. Advances are also needed in reliable management of depth and occlusion in mixed reality scenes, as well as adaptive lighting, color, and contrast adjustment.

### Non-Uniform Availability of Surgical Expertise Across Geographic Regions

Many countries suffer from a concentration of surgical expertise in a few main urban centers due to economic and political factors. At the same time, anticipated demographic shifts are expected to result in fewer health care professionals and more people in need of health care. XR technologies have the potential to facilitate access to medical expertise in short supply by conveying visual and haptic features in real time so that remote surgeons can actively participate in surgery. Realizing this will require advances in techniques to create a sense of presence, to general setups that support a variety of procedures, and reliable and low-latency networking and communication.

### Facilitating Minimally Invasive Surgery to Improve Outcomes

Surgery is continuing the trend to become increasingly minimally invasive due to the decreased trauma to the patient, resulting in better outcomes and reduced recovery time. Robot-assisted surgery has become essential in supporting the minimally invasive approach. It also enables manipulation of anatomical structures too small to be perceived and manipulated by non-augmented humans. XR technology has the potential to support robot-assisted minimally-invasive surgery by compensating for the limited field of view and providing accurate haptic feedback. Realizing this potential will require advances in real-time tracking and registration to support AR overlays and extremely low latency to support real-time feedback.

### Increasing the Effectiveness of Patient Participation in Decision Making

Surgeons are increasingly expected to consider not only the physical aspects of surgery but also the patient’s perspective. This includes providing them with an understanding of the procedure, its potential outcomes, any risks involved, as well as involving them in the decision process. Furthermore, efforts should be made to reduce patient anxiety that commonly arises when facing any surgery. Through immersive visualization, XR can provide pain modulation and emotional support during surgery. XR can also provide personalized immersive explanations that help to make the procedure and outcomes understandable and relatable and thus help reduce anxiety, as well as enabling the patient to make better informed decisions. The main challenge in realizing these benefits is less purely technical and more driven by psychology and perception. Essential questions include what kind of experience would best calm the patient and how best to convey what post-surgical life will be like, particularly since patients often go through some emotional adaptation to new states in life.

### Evaluation

Effective approaches to evaluation will be essential to moving forward on all the fronts we have just enumerated, but is a highly complex task. It naturally must consider technical performance aspects, but also much more. It needs to consider the perspectives of the surgeon, the health care system, the patient, and society. It must consider quality of care, as well as costs and take short-term and long-term perspectives. Evaluation for solutions will thus require multi-dimensional analysis. The objective remains the implementation of validated clinical environments that enhance procedural capabilities while maintaining safety and efficiency standards.

Realizing the full potential of XR in the OR will require concerted interdisciplinary research efforts, as well as coordinated multidimensional validation efforts. We hope this perspective paper has helped to illuminate the immense promise but also significant challenges to effectively applying XR in the OR and thus to map out some of the most important research problem and ultimately stimulate increased research in this important area that has such potential for positive impact.

## Data Availability

No datasets were generated or analysed during the current study.

## Footnotes

1
https://www.dagstuhl.de/25062.

2 See e.g. https://caresyntax.com/platform/, https://www.getinge.com/de/produkte/tegris/.

3
https://nebula.stream/needmi/index.html.

4 See apoQlar’s Holomedicine Spaces: https://apoqlar.com/spaces/.
